# Morton Code-Based Geometry-Adaptive Surface Reconstruction

**DOI:** 10.3390/jimaging12060225

**Published:** 2026-05-26

**Authors:** Zili Huang, Ran Fan, Yongwei Miao

**Affiliations:** School of Information Science and Technology, Hangzhou Normal University, Hangzhou 311121, China; randomhuangzili@163.com (Z.H.); ywmiao@hznu.edu.cn (Y.M.)

**Keywords:** surface reconstruction, Morton code, octree encoding, hierarchical feature encoding, adaptive weights

## Abstract

Neural implicit surface representations have yielded impressive results in 3D reconstruction, yet existing methods tend to introduce noise in smooth regions or fail to capture fine details in complex areas, primarily due to a lack of explicit spatial structure modeling. To address these limitations, we propose a geometry-adaptive surface reconstruction method based on Morton codes. By mapping 3D space onto octree traversal paths, this approach provides a natural spatial structural prior for the reconstruction process. For each query point, an implicit octree generates a unique root-to-leaf trajectory, yielding spatially adaptive weights that modulate multi-resolution geometric features. Specifically, low-frequency coarse features dominate in flat regions to suppress noise, whereas high-frequency fine features are activated in edge-rich areas to recover intricate geometry. Experimental results demonstrate competitive performance across multiple datasets, particularly in reconstructing sharp features and fine-grained geometric details.

## 1. Introduction

Three-dimensional reconstruction is a core research problem in computer vision and graphics, aiming to recover three-dimensional geometry from two-dimensional images. This technology has broad applications in augmented reality (AR), virtual reality (VR), digital twins, autonomous robotics, and cultural heritage preservation [1].

The emergence of neural implicit representations has recently brought a paradigm shift to 3D reconstruction. Unlike traditional explicit representations (e.g., voxels [2], point clouds [3], meshes [4]), neural implicit representations [5,6] model geometry as continuous functions via neural networks, enabling arbitrary-resolution representation of surfaces with complex topology. Neural Radiance Fields (NeRFs) [7] demonstrated impressive results in novel view synthesis through volume rendering. However, NeRF models scenes via volumetric density fields whose zero-level sets do not correspond to precise geometry, leading to noisy reconstructions that struggle to capture sharp edges and fine textures [8]. To overcome this, subsequent works such as NeuS [9] and VolSDF [10] integrated signed distance functions (SDFs) with volume rendering, defining surfaces as SDF zero-level sets and significantly improving reconstruction quality.

Despite these advances, neural implicit methods still face limitations in recovering complex surface details. The “spectral bias” of neural networks [11] causes them to prioritize low-frequency, smooth functions, while high-frequency geometric details (e.g., edges, textures) remain difficult to learn [12]. Positional encoding [13] was introduced to address this by mapping coordinates to high-dimensional spaces, but fixed-frequency sinusoidal encodings lack spatial adaptability: uniformly increasing frequency can cause instability and introduce noise in smooth regions. Subsequent work, such as SAPE [14], attempted to learn spatially adaptive frequencies; yet, its representational capacity remains limited.

As an alternative, feature grid-based encoding [15] has gained attention. These methods discretize space into grids storing learnable features, with interpolation at query points. Multi-resolution grids, such as Go-Surf [16], enable learning geometric features from coarse to fine scales. However, existing methods fuse all resolution features with fixed weights, failing to adaptively allocate representational capacity according to local geometric complexity. Consequently, smooth planes and complex textures are treated equally, leading to wasted computation and potential overfitting or underfitting. The deeper issue is that these methods treat 3D points as independent entities, querying features solely by coordinates, thus lacking awareness of spatial adjacency or macroscopic structural context. Unlike existing multi-resolution grid methods that fuse features from all levels with fixed weights, and unlike frequency-domain adaptive encoding methods that lack explicit spatial hierarchy modeling, our method introduces a path-aware adaptive weighting mechanism that explicitly leverages the octree traversal path. To address the challenges of spatially adaptive feature fusion, we propose a geometry-adaptive surface reconstruction method based on Morton codes. The core idea is to leverage octrees—which naturally encode spatial multi-scale hierarchies—and Morton codes—which linearize hierarchical paths—as a bridge connecting spatial structure to neural feature learning. The key novelty is not any single component, but the principled combination of Morton codes (to linearize the root-to-leaf path while preserving prefix dependency), a GRU-based recurrent encoder (to model cross-level dependencies along that path), and a progressive training schedule (co-designed with path activation). Specifically, we use Morton codes to establish coarse-to-fine octree traversal paths for each query point. We construct two parallel multi-resolution grids: a geometric feature grid storing implicit features, and a spatial mask grid storing mask features for fusion. A hierarchical prefix recurrent encoder aggregates context along the Morton path and predicts spatially adaptive fusion weights. These weights dynamically fuse multi-resolution features, which are then decoded into precise SDF values. Morton codes thus serve as a “spatial path index,” enabling learnable, spatially varying allocation of representational capacity.

The main contributions of this work are as follows:

- A multi-resolution spatially adaptive framework: By using Morton code-based mask grids, 3D spatial points are mapped onto octree paths that naturally encode multi-scale contextual information, enabling dynamic adjustment of 3D scene features and breaking through the limitations of traditional fixed-resolution or uniform fusion methods.
- A hierarchical prefix recurrent encoder is designed, which effectively aggregates hierarchical contextual information along octree paths, generating information-rich, path-aware feature representations for each query point. Compared to methods that directly concatenate or average features from different levels, this effectively captures inter-level dependencies.

## 2. Related Work

### 2.1. Neural Implicit Surface Reconstruction

Using neural networks as parametric forms of implicit functions to model geometric shapes has become mainstream. Early works such as Occupancy Networks [5] and DeepSDF [6] learned occupancy probabilities or distances to surfaces via fully connected networks, but they required 3D supervision. The pioneering work of NeRF [7] combined volume rendering with neural radiance fields, enabling optimization using only 2D images. However, NeRF represents scenes through volumetric density fields, whose zero-level sets do not correspond to precise geometric surfaces, resulting in reconstructed geometry often containing noise and artifacts.

Early methods, such as DVR [17] and IDR [18], directly optimized implicit surfaces through differentiable surface rendering. Although these methods accurately reconstruct geometric details, their optimization provides gradient information only near ray-surface intersections, thus heavily relying on precise pixel-wise object masks as supervision, limiting their applicability in mask-free scenarios. UNISURF [19] proposed a unified framework combining neural implicit surface representation with radiance fields, achieving reconstruction quality comparable to IDR without requiring object masks while significantly outperforming NeRF in geometric reconstruction.

NeuS [9] and VolSDF [10] further proposed a new paradigm combining SDF with volume rendering, achieving more accurate geometric modeling by defining surfaces as the zero-level sets of SDFs. NeuS introduced an unbiased occlusion-aware weight that enables volume rendering to directly optimize the SDF network, while VolSDF established theoretical connections between the two representations by converting SDFs to volumetric densities. Subsequent variants improved input feature encoders; for instance, HF-NeuS [20] introduced an additional MLP to learn high-frequency details, while PET-NeuS [21] incorporated tri-plane networks to extract additional detail features. NeuralWarp [22] added cross-view photometric consistency terms to neural rendering optimization, and GeoNeuS [23] theoretically analyzed the gap between volume rendering integration and point-based SDF modeling, introducing explicit multi-view geometric constraints to enhance photometric and depth consistency across views. Other methods enhanced robustness and detail by integrating prior information such as monocular depth [24], normal estimates [25,26], and RGB images [27]. Several approaches leverage specialized grid structures to accelerate training and improve surface reconstruction accuracy; for example, Vox-Surf [28] uses octrees to manage sparse grids, while Instant-NGP [15] and Co-SLAM [29] employ hash encoding as feature lookup tables for network inputs, significantly speeding up training.

### 2.2. Advanced Imaging and Data-Driven Methods

Beyond neural implicit representations and positional encoding, several recent studies in advanced imaging and data-driven reconstruction techniques offer complementary insights. First, RGB-polarization (RGB-P) imaging has emerged as a powerful modality for capturing both color and polarization information. Karge et al. [30] proposed a neural-network-based characterization method for RGB-P sensors. By measuring spectral responsivities for polarized irradiance and modeling chromaticity drift, their method enables accurate reconstruction of color and polarization features—an important capability for surface normal estimation and material analysis. Although polarization cues are not directly used in our current framework, such multi-modal imaging points to future integration of geometry-aware and material-aware reconstruction. Second, deep learning combined with clustering has been applied to 3D point cloud denoising and optimization. Emadi and Limongiello [31] introduced a two-step approach: a variational autoencoder reduces feature dimensionality, followed by clustering models (k-means, agglomerative, spectral, GMM) to identify and mitigate noise. Their evaluation of cultural heritage point clouds shows that k-means achieves the best cluster separation and accuracy. While our method focuses on implicit SDF modeling via octree-based grids, both approaches share the principle of hierarchical feature encoding and data-driven regularization.Third, for surface defect detection, Yang et al. [32] proposed DAER-YOLO, a lightweight model integrating a C3k2-iEMA module for defect-aware feature enhancement and an Efficient Up-Convolution Block (EUCB) for edge reconstruction. Their method improves mAP@50:95 by 2.3% on varistor surface defects, with particular gains on edge damage and small-object defects. The idea of emphasizing edge-rich regions aligns with our geometry-adaptive mechanism, which selectively activates high-frequency features in areas with intricate geometric details. Finally, from a methodological perspective, Khalife et al. [33] developed a process for constructing a Value Attributes List (VAL) in construction projects, involving stakeholder identification, iterative refinement, and adaptive weighting of competing values. Although not directly related to 3D reconstruction, this framework offers an analogy to our hierarchical multi-resolution feature fusion, highlighting the importance of adaptive weighting among different levels of abstraction. Collectively, these works illustrate the growing interest in multi-modal imaging, data-driven denoising, edge-aware detection, and adaptive weighting across different research communities.

### 2.3. Positional Encoding

Positional encoding is crucial for optimizing neural networks on low-dimensional domains, as neural networks tend to prioritize learning low-frequency functions and struggle to directly fit high-frequency details. Consequently, input coordinates typically need to be encoded into high-dimensional spaces. This phenomenon stems from theoretical analysis of the neural tangent kernel (NTK), which shows that the convergence speed of fully connected networks is negatively correlated with the frequency components of the target function [11]. Thus, mapping low-dimensional coordinates to high-dimensional feature spaces helps neural networks capture complex details and spatial relationships [13].

Fixed-frequency sinusoidal encoding was first popularized by NeRF. This encoding method, similar to Fourier feature mapping, lifts input coordinates into high-dimensional spaces using multiple sets of sine and cosine functions at different frequencies. Tancik et al. [13] theoretically proved that such Fourier feature mapping significantly accelerates network convergence and enhances the learning of high-frequency details. However, fixed-frequency encoding has inherent limitations: all spatial locations share the same set of frequencies, preventing the model from dynamically adjusting representational capacity according to local geometric complexity. Setting the frequency range too low makes it difficult to capture fine textures, while setting it too high may introduce high-frequency noise, and interference between different frequency components can cause optimization instability [14]. Subsequently, learnable Fourier features emerged as an improvement over fixed-frequency encoding, allowing networks to adaptively adjust frequency parameters during training. SIREN [34] proposed using sine as an activation function with a carefully designed initialization strategy, enabling networks to represent high-order derivatives of complex signals. In contrast, Fourier feature networks [13] place a learnable linear transformation layer before the input, enabling networks to learn task-optimal frequency distributions. Although learnable approaches offer greater flexibility, they still perform global frequency adjustment and fail to achieve spatially varying frequency allocation. To further address this limitation, SAPE [14] introduced a spatially adaptive progressive encoding framework, dynamically assigning different frequency components to different regions by learning a modulation network conditioned on spatial coordinates. During optimization, SAPE employs a progressive exposure strategy from low to high frequencies, allowing the model to first learn coarse geometric structures before gradually introducing high-frequency details. This coarse-to-fine optimization paradigm significantly improves training stability.

Feature grid-based encoding [16,35] has recently gained attention as an alternative to sinusoidal encoding. The core idea of these methods is to discretize space into regular grid structures, storing learnable feature vectors at grid vertices, with query points obtaining continuous feature representations through trilinear interpolation. Compared to continuous positional encoding, feature grids exhibit explicit spatial locality, allowing neighboring points to share similar features within the grid. Additionally, feature grids offer flexible dimensionality choices, enabling each grid vertex to store arbitrary-dimensional features that are convenient for subsequent network training. Much of the training effort in these methods lies in the feature grids themselves, allowing the use of shallower, more efficient MLPs for optimization, thereby improving training and inference times. Plenoxels [35] and DVGO [36] further simplified neural field representations by directly storing physical quantities such as density and color at sparse voxel grid vertices, completely eliminating MLP structures. Plenoxels employs adaptive voxel subdivision, automatically refining voxels in detail-rich regions to achieve higher resolution, while DVGO enhances representational capacity by storing density and color in separate grids and introducing post-activation mechanisms. These methods store learnable parameters directly in grids and optimize them through differentiable rendering, achieving ultra-fast training convergence. However, grid-based methods have a significant drawback: for high-resolution reconstruction, memory requirements grow cubically as voxel count increases with resolution. Some approaches attempt to prune grids using octrees [37] or sparse grids [35], but these methods require complex training schemes and their dynamic data structures are difficult to accelerate on GPUs. To address these issues, Instant NGP [15] uses hash tables to store feature vectors, mapping input coordinates via hash functions, enabling networks to learn compact scene representations under memory constraints. Instant NGP’s encoding accelerates training by several orders of magnitude while maintaining reconstruction quality comparable to high-quality NeRF.

In the field of surface reconstruction, Go-Surf [16] introduced a method combining multi-resolution feature grids with pooling mechanisms. Go-Surf leverages pooling operations between neighboring grid features to infer geometric information in unobserved regions, significantly improving reconstruction completeness and robustness. However, Go-Surf still employs a fixed feature fusion strategy, concatenating all resolution features with equal weights before input to the decoder, failing to dynamically adjust the contribution of each level according to local geometric complexity.

The geometry-adaptive surface reconstruction method based on Morton codes proposed in this paper addresses the limitations of existing neural implicit reconstruction methods in representational capacity allocation while enhancing reconstruction accuracy for geometric details in complex scenes. By introducing a spatially adaptive feature fusion mechanism based on octrees and Morton codes, we achieve dynamic allocation of geometric representational capacity. Specifically, our method first employs an octree structure to organize multi-resolution feature grids and utilizes Morton codes to efficiently map 3D spatial points onto coarse-to-fine hierarchical traversal paths, preserving spatial locality while explicitly encoding parent–child dependencies across different resolutions. Building upon this, we design a hierarchical prefix recurrent encoder that progressively aggregates contextual information along octree paths, capturing semantic relationships between levels and generating path-aware global feature representations. Simultaneously, we introduce a spatially adaptive mask network that dynamically predicts fusion weights for features at different levels based on the aggregated features, enabling the model to favor low-frequency, coarse features in flat regions while activating high-frequency, fine-grained features to recover intricate geometric details in edge-rich and texture-complex areas.

## 3. Method

**Figure 1:**
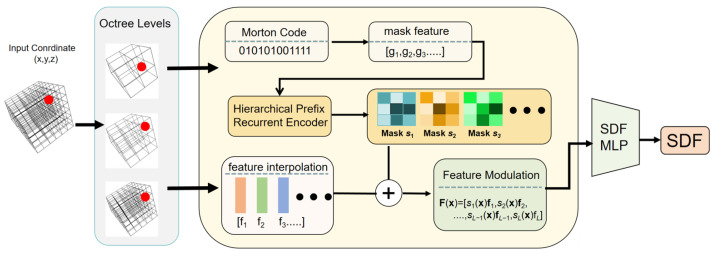
Framework of the Morton code-based geometry-adaptive surface reconstruction. A multi-resolution octree is constructed for hierarchical scene representation. Each query point is mapped via Morton code to a root-to-leaf octree path, along which the primary feature grid and a mask grid are queried. A hierarchical prefix recurrent encoder aggregates mask features from coarse to fine levels to generate spatially adaptive weights that dynamically fuse multi-resolution geometric features according to local geometric complexity.

Most existing neural implicit representation methods rely on fixed or global multi-resolution encoding strategies, such as multi-resolution grids or fixed-frequency sinusoidal encoding. In these methods, features from all resolution levels are treated equally and used for decoding every spatial point, without considering the local geometric characteristics of that point. However, an ideal representation should be adaptive: in geometrically complex, detail-rich regions (e.g., object edges, hollow structures), higher-resolution features are needed for precise representation; while in flat, smooth regions (e.g., walls, tabletops), lower-resolution features suffice for accurate representation, and introducing high-frequency information may instead cause artifacts and noise. To achieve such adaptivity, a data structure that can efficiently encode spatial multi-scale hierarchies and adjacency relationships is first required. Octrees naturally represent coarse-to-fine hierarchical structures through recursively subdivided voxels, while Morton codes provide a compact indexing mechanism that linearizes octree paths—by interleaving the binary bits of coordinates, Morton codes map 3D coordinates onto a one-dimensional Z-order curve, preserving spatial locality while explicitly encoding the root-to-leaf traversal path of each node in the octree. To leverage these properties, we propose a framework as illustrated in Figure 1.

### 3.1. Multi-Resolution Octree Representation

**Figure 2:**
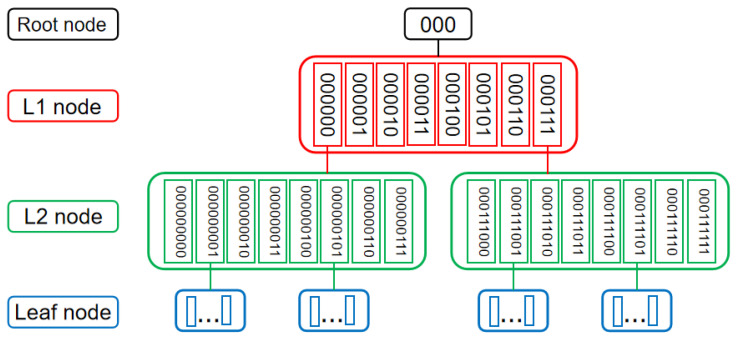
Illustration of octree structure and Morton code nodes.

To achieve spatially adaptive features, we divide the octree grid into two components. The first is the geometric and color feature grid, which stores the implicit representation of objects. The second is a smaller mask grid, which stores spatially varying mask features used to adjust the encoding of the SDF. As shown in Figure 2, we store scene features in a sparse grid system controlled by an L-level octree. Meanwhile, we assign each grid cell an interlaced binary Morton code (three bits per level, representing the relative positions along the x, y, and z axes, respectively). This unique code encapsulates both the global position information of the grid cell and the relative information between parent and child nodes. For a given query point x=(x,y,z), our method obtains the feature vector f from the multi-resolution geometric feature grid via trilinear interpolation, and returns the feature vector g from the spatial mask grid.

To efficiently index the octree structure, we adopt Morton codes (Z-order curves) to linearize 3D spatial coordinates. For a discrete grid coordinate $\left(x,y,z\right)\in {\left[0,2^{L}\right)}^{3}$, its binary expansions are given by $x=(x_{L-1},\ldots x_{0})$, $y=(y_{L-1},\ldots y_{0})$, $z=(z_{L-1},\ldots z_{0})$. The Morton code is constructed by interleaving the binary bits of the three coordinates:

(1) $$M\left(x,y,z\right)=\left(x_{L-1},y_{L-1},z_{L-1},x_{L-2},y_{L-2},z_{L-2},\ldots ,x_{0},y_{0},z_{0}\right)$$

**Figure 3:**
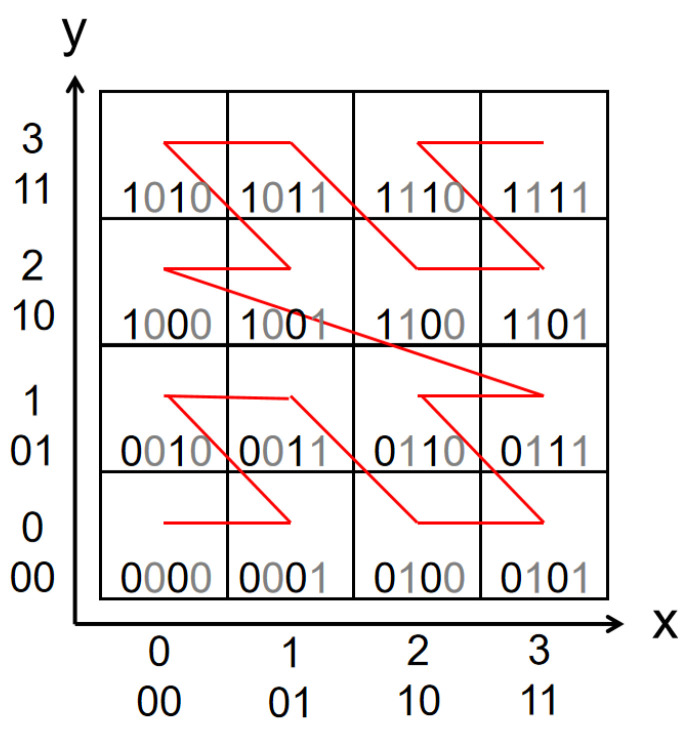
Illustration of Morton code (Z-order curve).

Figure 3 shows an example of a 2D Morton curve filling. The Morton encoding process converts a 3D coordinate into a one-dimensional code while largely preserving spatial locality. Moreover, Morton codes effectively encode the hierarchical traversal paths of the octree. For a spatial point, the relative position at each octree level can be represented by a three-bit code $\left(x_{i},y_{i},z_{i}\right)$, and the corresponding child index in the octree grid at level i is given by:

(2) $$\Delta_{i}=x_{i}+2y_{i}+4z_{i}$$

where $\Delta_{i}\in \left\{0,\ldots 7\right\}$. Thus, the Morton code can be decomposed as $\Delta \left(x\right)\in \left\{\Delta_{1},\ldots \Delta_{L}\right\}$, which describes the coarse-to-fine octree traversal path. The Morton code encodes the octree hierarchical path of a 3D point into a compact sequence that indicates the child node index at each level and implicitly conveys multi-scale spatial context—points sharing the same prefix (i.e., same ancestor) have similar positions at coarse scales. This path representation offers three theoretical advantages over alternatives. First, locality preservation: Morton codes map nearby 3D points to nearby 1D codes with bounded distortion, while requiring only O(1) bitwise operations, unlike Hilbert curves, which need expensive recursive encoding. Second, exact hierarchical alignment: The natural decomposition into 3-bit prefixes $\Delta_{1},\ldots ,\Delta_{L}$ provides a bijective mapping to the octree traversal path, enabling a recurrent encoder to process coarse-to-fine steps explicitly. Third, coding efficiency: The prefix property (parent code = prefix of child code) allows computation sharing across levels and compresses the entire path into a single 3L-bit integer, whereas alternative curves lack this property. Therefore, based on the Morton code path representation, our network perceives multi-scale spatial context and feeds the hierarchical prefix sequence into a prefix recurrent encoder to generate spatially adaptive fusion weights.

### 3.2. Hierarchical Prefix Recurrent Encoding

**Figure 4:**
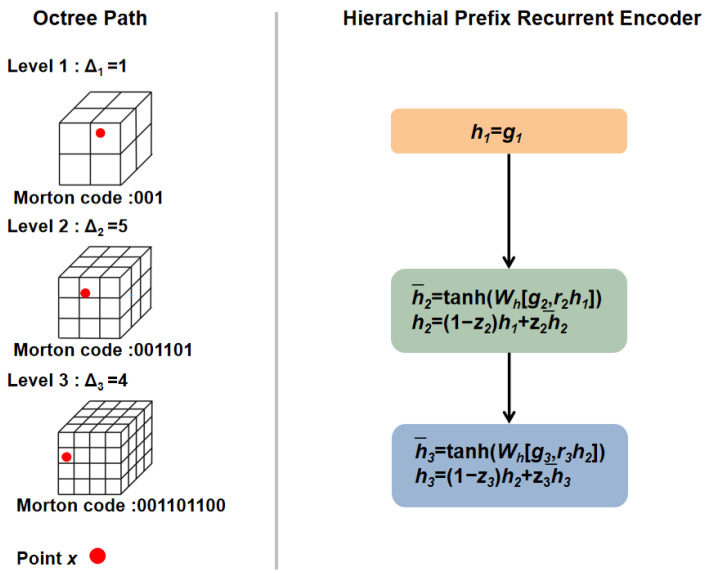
Illustration of hierarchical prefix recurrent encoding.

To capture the hierarchical and local spatial information embedded in the octree grid, as illustrated in Figure 4, Morton codes naturally encode hierarchical structures. Except for the last three bits, the code of a child node shares the same prefix as its parent, meaning the parent’s code can be regarded as the prefix of the child’s code. We argue that deep features should be interpreted based on shallow context, and features at different levels are both interrelated and specialized: shallow grids capture large-scale geometric structures, while deep grids recover fine details. To effectively model this hierarchical dependency while progressively introducing frequency information during training, we propose a hierarchical prefix recurrent encoder based on a gated recurrent unit (GRU). This encoder recursively aggregates contextual information along the octree path from coarse to fine levels, generating masks at each step and controlling the retention and update of historical information through a gating mechanism. Meanwhile, we gradually expose deeper-level grids during training and freeze already converged low-frequency features in later stages, thereby achieving orderly learning of frequency information and stable optimization.

Mask features at different levels are not independent; they form a hierarchical structure with dependencies along the octree path. To model these dependencies, we use an encoder to recursively process the sequence of mask features, aggregating information from coarse to fine levels. The mask feature at the first level is initialized as $h_{1}=g_{1}$. For each subsequent level l, we adopt a GRU-style update mechanism:

(3) $$z_{l}=\sigma \left(W_{z}\left[g_{l},h_{l-1}\right]\right)$$

(4) $$r_{l}=\sigma \left(W_{r}\left[g_{l},h_{l-1}\right]\right)$$

(5) $$\bar{h_{l}}=tanh\left(W_{h}\left[g_{l},r_{l}⊙h_{l-1}\right]\right)$$

(6) $$h_{l}=\left(1-z_{l}\right)⊙h_{l-1}+z_{l}⊙\bar{h_{l}}$$

where σ denotes the sigmoid activation function, and $W_{z},W_{r},W_{h}$ are learnable weight matrices. The choice of the GRU as the hierarchical prefix recurrent encoder is primarily justified by two considerations. First, GRU naturally models the hierarchical dependencies along Morton code paths: the information at the current level inherently depends on all coarser-level history. The GRU explicitly accumulates historical information via its hidden state $h_{l-1}$ and adaptively fuses the current input $g_{l}$ through a gating mechanism, which perfectly aligns with the “prefix dependency” property of octree traversal paths. In contrast, MLPs treat all level features as a flat concatenation, failing to capture sequential order dependencies; one-dimensional convolutions can only exploit local context within a limited receptive field and struggle to model long-range cross-scale dependencies. Second, GRU offers favorable parameter efficiency and training stability. It is a lightweight recurrent architecture whose parameter count is significantly lower than that of a self-attention model with the same hidden dimension. In our setting, the path length L is typically no more than 10, making the unrolled recurrent steps short and mitigating the risk of vanishing gradients. Moreover, the update and reset gates of the GRU effectively control information flow, preventing early coarse features from being overwhelmed by later fine-grained features. We use a single-layer GRU because the dependencies along the octree path are strictly layer-by-layer progressive; a multi-layer GRU does not bring additional benefits and may increase the risk of overfitting. This residual recursive formulation progressively aggregates spatial context along the Morton-encoded path. At each level, the representation is based on the current cell features and the context accumulated from coarser levels. Ultimately, the mask feature $h_{L}$ at the final level encodes the complete hierarchical context of the query point. This feature is then used to generate the spatial mask *s*:

(7) $$s=softmax(f_{s}\left(h_{L}\right))$$

where $f_{s}$ is a shallow multi-layer perceptron, and the mask *s* is an L-dimensional vector with $s_{l}\in \left(0,1\right)$ for each level *l*. This mask acts as a spatial function, enabling the network to adaptively adjust the contribution of each resolution grid to the reconstruction result—for instance, enhancing the contribution of low-frequency grids in smooth regions while preserving high-frequency features in detail-rich areas. The multi-resolution geometric feature vectors at the query point, denoted as $f=[f_{1},f_{2},\ldots ,f_{L}]$, are obtained via trilinear interpolation from the multi-resolution grids. Combined with the spatial mask *s*, the final Morton-encoded feature is computed as:

(8) $$F\left(x\right)=[s_{1}\left(x\right)f_{1},s_{2}\left(x\right)f_{2},\ldots ,s_{L}\left(x\right)f_{L}]$$

The spatial mask function *s* dynamically adjusts the contribution weights of features at each resolution level based on the query point’s location. In flat regions, the network tends to assign higher weights to low-resolution features while suppressing high-resolution features, thereby avoiding overfitting to noise. In geometrically complex regions, the weights of high-resolution features are activated, enabling the model to capture fine geometric variations. This spatial variation of weights is precisely generated by the Morton code-based hierarchical recurrent encoder, achieving “geometry-adaptive” representational capacity.

To stably learn multi-scale geometric features during optimization, we adopt a progressive encoding strategy. This strategy gradually exposes deeper octree nodes (i.e., higher-resolution features) to the network, guiding it to learn from coarse geometric structures and progressively refine to fine details. Specifically, we define the maximum depth of the octree as *L*, corresponding to *L* resolution levels from low to high. In the early stages of training, only nodes at shallower depths are activated, allowing the network to primarily learn low-frequency, smooth geometric shapes. As training progresses, we gradually increase the maximum activation depth, adding one level every certain number of iterations until the maximum depth *L* is reached. In practice, we adopt a simple epoch-based stagewise activation schedule. The total number of training epochs is 4000, and the octree has a maximum depth of L = 9. During the first 500 epochs, only the four coarsest octree levels (levels 1–4) are activated. From epoch 500 to 1500, levels 5–6 are additionally activated. From epoch 1500 to 3000, levels 7–8 are added. Finally, from epoch 3000 to 4000, the finest level (level 9) is activated. For feature layers that have not yet been activated, we block gradient backpropagation to their corresponding spatial masks by preventing the trainable parameters of those layers from receiving any gradient updates in the optimizer. This prevents the mask network from prematurely deciding to suppress high-resolution features that will be needed for fine reconstruction later, preserving their availability during the later stages of training.

### 3.3. Volume Rendering

For the octree-based multi-resolution grid system proposed in this paper, we adopt a flexible hierarchical independent adaptive sampling and rendering strategy. In a multi-resolution sparse grid system, traditional multi-sampling mechanisms waste significant computational time in regions without surfaces. Therefore, we achieve refined sampling control by setting differentiated sampling parameters for grids at different levels. For each sampling ray, we first detect its intersection with the grid. Due to the octree structure, a coarse-to-fine ray–grid intersection path can be efficiently computed. Based on the intersection points between the ray and grids at different levels, we compute the intersection intervals for each level. For each grid level, we employ inverse transform sampling according to the length of the intersection interval on that level, ensuring that more sampling points are allocated to levels with longer intersection intervals. Meanwhile, different sampling step sizes are set for different grid levels, with smaller step sizes for higher-resolution grids to achieve denser sampling. This approach ensures that the sampling point distribution matches the geometric importance of the grids. For any query point *x*, its complete geometric feature F(x) is fed into the SDF decoder to predict the signed distance value:

(9) $$\phi \left(x\right)=f_{sdf}\left(F\left(x\right)\right)$$

For color, an independent color feature $F_{r}gb\left(x\right)$ and decoder $f_{rgb}\left(\cdot \right)$ are used:

(10) $$c\left(x\right)=f_{rgb}\left(F_{rgb}\left(x\right)\right)$$

We adopt a rendering formulation similar to Neural RGB-D [38], replacing the original global coordinates with voxel embeddings. The signed distance ϕ(x) is converted into a weight $w_{n}$ for color and depth accumulation along the sampling ray in volume rendering:

(11) $$w_{n}=\sigma \left(\frac{\phi (x)}{tr}\right)\cdot \sigma \left(-\frac{\phi (x)}{tr}\right)$$

where σ(·) is the sigmoid function and tr is a preset truncation distance. The final color and depth values are obtained via weighted integration along the ray:

(12) $$C=\frac{1}{\sum_{n=0}^{N-1}w_{n}}\sum_{n=0}^{N-1}w_{n}\cdot c(x)$$

(13) $$D=\frac{1}{\sum_{n=0}^{N-1}w_{n}}\sum_{n=0}^{N-1}w_{n}\cdot d_{n}$$

### 3.4. Loss Function

To optimize our scene representation, we sample a batch of rays from all pixels of all images in each iteration. Our global loss function L is defined as:

(14) $$L=\lambda_{d}\cdot L_{d}+\lambda_{rgb}\cdot L_{rgb}+\lambda_{sdf}\cdot L_{sdf}+\lambda_{fs}\cdot L_{fs}+\lambda_{eik}\cdot L_{eik}+\lambda_{curv}\cdot L_{curv}$$

$L_{rgb}$ and $L_{d}$ compare the differences between the ground-truth colors, depths, and the rendered values. $L_{rgb}$ is computed over all sampled rays, while for $L_{d}$, only rays with valid depth values $R_{d}$ are considered. The L1 loss is adopted for both color loss and depth loss:

(15) $$L_{rgb}={\left|I\left[u,v\right]-C\right|}_{L1}$$

(16) $$L_{d}={\left|D\left[u,v\right]-D\right|}_{L1}$$

For the rendering-supervised signed distance loss, at the sampled points along each ray, the depth value along the ray direction is used to approximate the SDF value near the object surface, that is $\varphi_{gt}\left(x\right)=D\left[u,v\right]-z$, where *z* is the depth of sample point x along the ray. Within the truncation region (|D[u,v]−z|<tr), the following loss is applied:

(17) $$L_{sdf}\left(x\right)={\left(\varphi \left(x\right)-\varphi_{gt}\left(x\right)\right)}^{2}$$

The free-space loss is applied to points far from the surface (|D[u,v]−z|>tr), that is:

(18) $$L_{fs}\left(x\right)={\left(\varphi \left(x\right)-tr\right)}^{2}$$

$L_{eik}$ is applied to points far from the surface (|D[u,v]−z|>tr), ensuring they also yield valid signed distance predictions:

(19) $$L_{eik}=\frac{1}{N}\sum_{i=1}^{N}{\left(||\nabla \varphi (x_{i})|{|}_{2}-1\right)}^{2}$$

Additionally, we introduce a curvature loss to enhance smoothness. The mean curvature is computed using a discrete Laplace operator, similar to the computation of surface normals:

(20) $$L_{curv}=\frac{1}{N}\sum_{i=1}^{N}\left|\nabla^{2}\varphi (x_{i})\right|$$

The spatial masks and feature networks are jointly trained in an end-to-end manner. The final weights are set as $\lambda_{rgb}=10.0$, $\lambda_{d}=1.0$, $\lambda_{sdf}=10.0$, $\lambda_{fs}=1.0$, $\lambda_{eik}=1.0$, $\lambda_{curv}=1.0$.

## 4. Experiment

The experiments in this paper were conducted in the following environment: CPU (Intel Corporation, Santa Clara, CA, USA) 2.50 GHz Intel^®^ Core™ i5-12400, 16 GB RAM, GPU (NVIDIA Corporation, Santa Clara, CA, USA) GeForce RTX 3060 with 12 GB VRAM. The operating system is Ubuntu 20.04 (Canonical Ltd., London, UK), and the software development platform is Python 3.9 (Python Software Foundation, Beaverton, OR, USA) and PyTorch 2.1.2 (Meta Platforms, Inc., Menlo Park, CA, USA). During training, we sample M = 6144 rays per batch. For each ray, we perform octree traversal to compute ray-voxel intersections, with a maximum of 50 voxels per ray. Sample points are then drawn along the ray using inverse transform sampling with a step size of half the voxel size, where the sampling probability of each voxel is proportional to its depth interval length. The finest resolution of our multi-resolution grid is $512^{3}$. The geometric feature dimension per level is 4, the mask feature dimension is 16, the GRU hidden state dimension is 32, and the color feature dimension is 6. For both geometry and color decoders, we use light-weight MLPs with two hidden layers, each having 32 neurons. Depending on the geometric complexity of the object, the optimization process takes approximately 20–40 min, with GPU memory usage ranging from 5 to 8 GB.

### 4.1. Datasets and Evaluation Metrics

The experiments were conducted using the DTU dataset [39] and the 3D scanning dataset [40]. The 3D scanning dataset consists of scan results from various real-world objects. This dataset includes 29 common object categories, with multi-view images and pose data at a resolution of 2448 × 2048. The dataset covers various items such as artworks, machinery, toys, and kitchen utensils, and it outperforms similar datasets in terms of completeness and accuracy of depth data. The object surfaces are reconstructed by extracting the zero level set of the signed distance function using the Marching Cubes algorithm. The DTU dataset is a real-world dataset containing multi-view images and pose data at a resolution of 160 × 120.

### 4.2. Baseline Models

We compare our method with the following approaches: 1. sPSR [41], a classic point cloud geometric surface reconstruction method used to generate a basic reference model. 2. Go-Surf [16], which stores the scene using a multi-layer voxel grid and learns features by directly optimizing the grid. 3. Vox-fusion [42], which stores the scene using an octree-based sparse voxel grid; although it uses an octree, it does not extract local feature information from it for network training.

For the DTU dataset, following the official recommendations, the Chamfer Distance is used for evaluation. For the 3D scanning dataset, accuracy, completeness, Chamfer Distance, normal consistency, and F-score are used as metrics to evaluate the reconstruction results.

### 4.3. Results and Analysis

**Figure 5:**
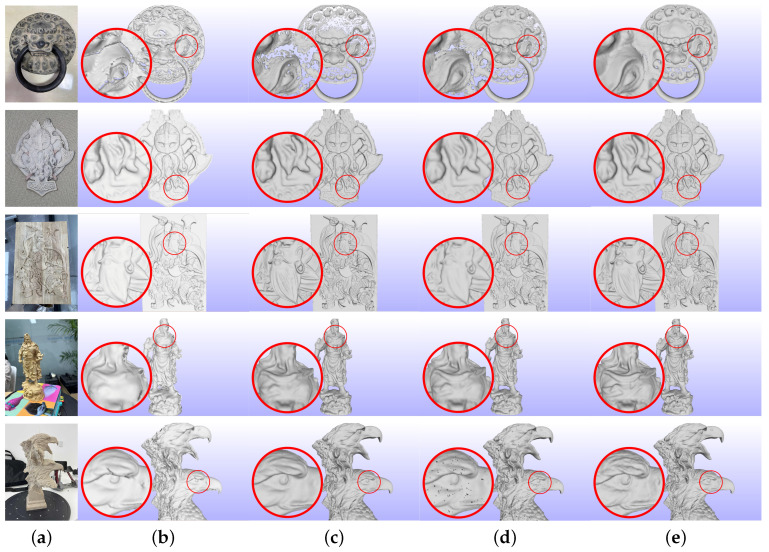
Comparison of reconstruction results on the 3D Scanning Dataset. (**a**) Image; (**b**) sPSR [41]; (**c**) Go-Surf [16]; (**d**) Vox-fusion [42]; (**e**) ours.

We first present the quantitative and qualitative comparison results on the 3D scanning dataset. Table 1 reports the quantitative metrics of our method and other methods across eight scenes. It can be observed that our method achieves the best performance across all scenes, demonstrating that the reconstruction results produced by our method have better completeness and accuracy. Meanwhile, the superior normal consistency indicates that our method more effectively recovers local details. In addition to outperforming other baseline methods in quantitative metrics, we also show the visual reconstruction results (Figure 5). It can be seen that in the reconstruction of geometric textures on the artworks and reliefs shown in the first three rows, such as beard and pattern details, our method reconstructs texture details more clearly and accurately. In the geometric texture reconstruction of sculptures and reliefs, our method produces finer texture details; for example, in the beard texture of the relief sculpture, the reconstruction results of other methods exhibit some degree of noise in this region, while in the eagle-head sculpture, our method achieves clearer details in the nostrils and feathers. Furthermore, in the door handle scene shown in the first row, significant occlusions caused by scanning angles are present; yet, our method achieves more complete reconstruction results in these regions compared to other methods.

**Figure 6:**
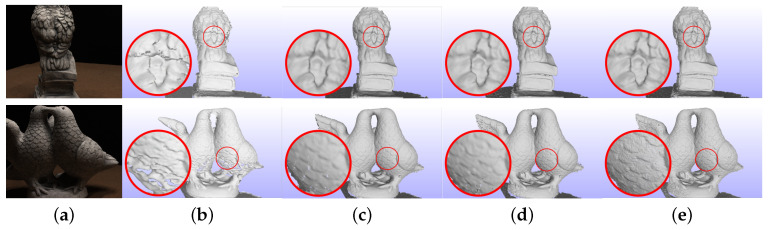
Comparison of reconstruction results on the DTU Dataset. (**a**) Image; (**b**) sPSR [41]; (**c**) Go-Surf [16]; (**d**) Vox-fusion [42]; (**e**) ours.

Quantitative results on the DTU dataset are shown in Table 2. Our method achieves superior Chamfer distance compared to all other competing methods on the DTU dataset. Qualitative comparisons with other methods are presented in Figure 6. The surfaces reconstructed by our method preserve finer details, such as the feather patterns on the owl and pigeon sculptures.

**Figure 7:**
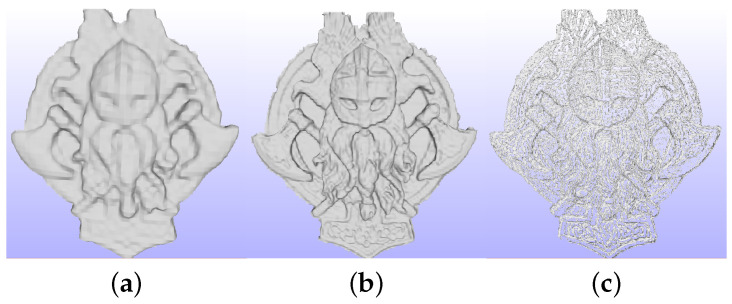
Individual reconstruction results of features at different levels. (**a**) level 5; (**b**) level 7; (**c**) level 9.

In addition, Figure 7 shows the reconstruction results obtained by independently decoding features from selected grid levels. The reconstruction from coarser-resolution grids (Figure 7a) fully preserves the macro-structure and topological continuity of the object, but exhibits over-smoothing at the detail level. The reconstruction from medium-resolution grids (Figure 7b) recovers some medium-scale features while maintaining the overall shape. The reconstruction from finest-resolution grids (Figure 7c) specifically captures fine textures and edge features, yet fails to ensure surface completeness when used alone. This highlights the importance of the spatially adaptive mask network in locally modulating features across different grid levels.

**Figure 8:**
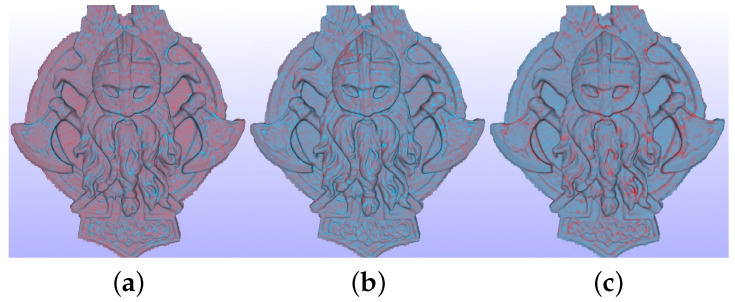
Visualization of spatially adaptive masks at different levels. (**a**) level 5; (**b**) level 7; (**c**) level 9.

Figure 8 presents the visualization of the spatially adaptive masks generated by our method. The masks at octree levels 5, 7, and 9 are selected for visualization, where colors closer to red indicate higher weights and colors closer to blue indicate lower weights. The mask at level 5 exhibits significantly higher weights in flat regions, while the mask at level 9 shows higher weights in regions with rich edges and geometric textures. A horizontal comparison clearly reveals that as the grid resolution increases, the mask weights in flat regions gradually decrease (transitioning from red to blue), whereas the weights in geometrically textured regions gradually increase (transitioning from blue to red).

To validate the effectiveness of each component in the proposed Morton code-based geometry-adaptive surface reconstruction method, we conducted a series of ablation experiments. Different model variants were quantitatively evaluated using metrics, including accuracy, completeness, Chamfer distance, normal consistency, and F-score. Table 3 presents the reconstruction results of four configurations: the variant without a spatial mask (w/o Mask), the variant with masks directly generated by an MLP (MLP-Mask), the variant that replaces Morton code with Hilbert curve (w/ Hilbert), and our full model (Ours, based on Morton code).

Experimental results show that introducing masks generated by a simple MLP leads to moderate improvements in accuracy, completeness, and Chamfer distance, confirming the effectiveness of the spatial adaptive mask mechanism. However, masks produced solely by an MLP struggle to adequately model the hierarchical dependencies among multi-resolution features, resulting in limited capability to modulate the contributions of different grid levels. In contrast, the full model leverages path-aggregated features extracted by the prefix encoder, providing the decoder with richer multi-scale structural priors and thus enabling more accurate spatially adaptive weights. Compared to the MLP-Mask variant, the full model achieves an 18.6% improvement in Chamfer distance and a 1.68% improvement in normal consistency, demonstrating the critical role of path-based encoding and hierarchical recurrent aggregation in enhancing reconstruction accuracy and detail fidelity.

Furthermore, we replaced Morton code with the Hilbert curve, which theoretically offers stronger locality preservation, to investigate the impact of different encoding schemes on reconstruction performance. Under the same network architecture and training settings, the Hilbert variant achieved results very close to those of the full model, with no statistically significant difference in accuracy. However, Hilbert encoding incurs significantly higher computational cost: in an encoding test with 100,000 random points, the Hilbert curve took 1.3195 s, while Morton encoding required only 0.0079 s, making Hilbert approximately 160 times slower. Moreover, Hilbert encoding does not directly represent octree parent–child relationships in its code, requiring additional decoding to fit our hierarchical recurrent encoder. Therefore, while maintaining reconstruction quality, we choose Morton code as the default encoding scheme due to its higher computational efficiency and natural alignment with octree structures.

## 5. Conclusions

To address the limitations of existing neural implicit surface reconstruction methods, where multi-resolution encoding lacks spatial adaptivity and struggles to dynamically allocate representational capacity according to local geometric complexity, this paper proposes a geometry-adaptive surface reconstruction method based on Morton codes. The method constructs an adaptive reconstruction framework guided by spatial structure priors: octrees and Morton codes are used to map 3D space into hierarchical traversal paths, providing the network with natural multi-scale spatial structure priors; a hierarchical prefix recurrent encoder aggregates contextual information along octree paths from coarse to fine levels, modeling dependencies between levels; based on the aggregated path features, spatially adaptive masks are generated to dynamically adjust the contribution of geometric features at each resolution to SDF decoding, thereby suppressing high-frequency noise in flat regions while activating fine details in geometrically complex areas. A progressive training strategy is also introduced, enabling the model to progress from low-frequency structures to high-frequency details, ensuring optimization stability and convergence quality.

Limitations. First, boundary artifacts occasionally appear near octree cell boundaries where mask weights change abruptly, manifesting as subtle surface discontinuities. Second, despite the use of octree-based sparse grid representation, the number of nodes still grows rapidly as the octree depth increases, leading to significantly higher memory consumption and computational overhead. Third, our current progressive training strategy relies on a manually predefined, epoch-based activation schedule rather than adapting to the local geometric complexity of the scene.

Future work can be pursued in the following directions. In terms of model efficiency, we will further optimize the octree storage structure and introduce node pruning strategies based on curvature or gradient magnitude to dynamically remove redundant nodes while preserving reconstruction quality, thereby reducing memory consumption and computational overhead. For dynamic scene modeling, we will investigate how to incorporate temporal information and motion estimation into the current framework to extend its applicability to non-rigid moving objects, enabling high-fidelity reconstruction of dynamic scenes. In the area of multi-modal fusion, we will combine neural rendering techniques to jointly optimize geometry and appearance, introduce physically based rendering models, and enhance reconstruction robustness for challenging materials such as specular surfaces and transparent objects, further expanding the method’s applicability to unconstrained real-world scenarios. 

## Figures and Tables

**Table 1:** Quantitative evaluation comparison of different methods on the 3D Scanning Dataset.

	Method	Artwork014	Artwork017	Artwork028	Artwork101	Fitting049	Sculpture011	Sculpture028	Sculpture051
Acc (↓)	sPSR	0.6728	0.7177	0.7172	0.5959	0.7801	0.7654	0.7812	0.7293
	Go-Surf	0.5166	0.5146	0.5245	0.7541	0.7536	0.5624	0.6312	0.5541
	Vox-fusion	0.5479	0.5254	0.5337	0.6461	0.7949	0.5747	0.6372	0.6024
	Ours	**0.4868**	**0.4951**	**0.4946**	**0.5215**	**0.6443**	**0.5283**	**0.5017**	**0.5004**
Com (↓)	sPSR	0.6494	1.7225	0.5371	0.5790	0.5640	0.6747	0.5715	0.5356
	Go-Surf	0.5133	0.7851	0.5385	0.5184	0.5139	0.5416	0.5174	0.5256
	Vox-fusion	0.5179	2.9373	0.9127	0.5580	0.5310	0.5644	0.5308	1.2160
	Ours	**0.4891**	**0.6543**	**0.4911**	**0.4964**	**0.5025**	**0.5268**	**0.5048**	**0.4916**
C-l1 (↓)	sPSR	0.6611	1.2201	0.6272	0.5875	0.6721	0.7200	0.6764	0.6324
	Go-Surf	0.5149	0.6499	0.5315	0.6363	0.6337	0.5520	0.5743	0.5399
	Vox-fusion	0.5328	1.7313	0.7232	0.6021	0.6630	0.5695	0.5840	0.9092
	Ours	**0.4879**	**0.5747**	**0.4928**	**0.5090**	**0.5734**	**0.5275**	**0.5033**	**0.4960**
Nc (↑)	sPSR	0.9040	0.9074	0.9254	0.9236	0.9306	0.9166	0.8884	0.9249
	Go-Surf	0.9777	0.9594	0.9685	0.9441	0.9541	0.9803	0.9642	0.9739
	Vox-fusion	0.9730	0.9366	0.9590	0.9220	0.9209	0.9750	0.9565	0.9446
	Ours	**0.9834**	**0.9789**	**0.9853**	**0.9568**	**0.9607**	**0.9825**	**0.9792**	**0.9811**
F-score (↑)	sPSR	0.8716	0.8313	0.8813	0.9199	0.8558	0.8516	0.8559	0.8807
	Go-Surf	0.9486	0.9215	0.9396	0.9063	0.8863	0.9405	0.9336	0.9390
	Vox-fusion	0.9327	0.6270	0.8420	0.9200	0.8658	0.9392	0.9263	0.7147
	Ours	**0.9537**	**0.9353**	**0.9581**	**0.9493**	**0.9125**	**0.9489**	**0.9589**	**0.9562**

Notes: ↓ indicates lower values are better; ↑ indicates higher values are better. Bold values denote the best performance per metric.

**Table 2:** Quantitative evaluation comparison of different methods on the DTU Dataset.

Method	4	20	46	53	99	103	106	114	118	123
sPSR	1.279	0.6873	0.8634	0.7947	0.8962	0.8721	1.3677	0.8957	2.6231	1.0676
Go-Surf	0.6851	0.7355	0.7814	0.7164	0.7905	0.7196	1.2641	0.6978	0.7107	0.7403
Vox-fusion	0.8445	0.8459	0.9107	0.9263	0.8565	0.8294	1.5081	0.9033	0.8755	0.9153
Ours	**0.6393**	**0.5913**	**0.5841**	**0.6175**	**0.6278**	**0.6085**	**0.9273**	**0.5791**	**0.5884**	**0.5783**

**Table 3:** Quantitative results of ablation experiments.

	Acc (↓)	Com (↓)	C-l1 (↓)	NC (↑)	F-Score (↑)
w/o Mask	0.7614	0.5461	0.6537	0.9412	0.9062
MLP-Mask	0.7167	0.5312	0.6239	0.9428	0.9109
w/ Hilbert	0.5232	0.4885	0.5059	0.9604	0.9445
Ours (Morton)	0.5244	0.4906	0.5075	0.9587	0.9428

Notes: ↓ indicates lower values are better; ↑ indicates higher values are better.

## Data Availability

The data presented in this study are openly available in DTU Robot Image Data at https://roboimagedata.compute.dtu.dk/?page_id=36 (accessed 20 May 2026).
